# Correction: Activity dynamics of amygdala GABAergic neurons during cataplexy of narcolepsy

**DOI:** 10.7554/eLife.52397

**Published:** 2019-10-04

**Authors:** Ying Sun, Carlos Blanco-Centurion, Emmaline Bendell, Aurelio Vidal-Ortiz, Siwei Luo, Meng Liu

Sun Y, Blanco-Centurion C, Bendell E, Vidal-Ortiz A, Luo S, Liu M. 2019. Activity dynamics of amygdala GABAergic neurons during cataplexy of narcolepsy. *eLife* **8**:e48311. doi: 10.7554/eLife.48311

Published 14, August 2019

Carlo Blanco-Centurion, Siwei Luo, and Aurelio Vidal-Ortiz’s contributions were originally listed in the “Acknowledgments” section. We believe that their contribution was underestimated and that they should have been included as authors. For example, after revisiting their contributions Dr. Blanco-Centurion had in fact additionally contributed to revising the manuscript whereas Dr. Luo and Mr. Vidal-Ortiz had also additionally participated in some of the animal surgery and data processing. We believe they meet the criteria for authorship and all authors agree they should be included in the author list.

Revised author list:

Ying Sun, Carlos Blanco-Centurion, Emmaline Bendell, Aurelio Vidal-Ortiz, Siwei Luo, Meng Liu

Original author list for reference:

Ying Sun, Emmaline Bendell, Meng Liu

Author details for the omitted authors:

**Carlos Blanco-Centurion**

Department of Psychiatry and Behavioral Sciences, Medical University of South Carolina, Charleston, United States

Contribution: Conceptualization, Methodology, software, Writing—review and editing

Competing interests: No competing interests declared

**Aurelio Vidal-Ortiz**

Department of Psychiatry and Behavioral Sciences, Medical University of South Carolina, Charleston, United States

Contribution: Methodology, Writing—review and editing

Competing interests: No competing interests declared

**Siwei Luo**

Department of Psychiatry and Behavioral Sciences, Medical University of South Carolina, Charleston, United States

Contribution: Methodology, Writing—review and editing

Competing interests: No competing interests declared

Revised Acknowledgements:

We thank Dr. Priyattam Shiromani for the advice, guidance, and technical support that made it possible to complete the study. We thank him for providing the miniscope and GRIN lenses for this study. Supported by NIH grants 1R01NS096151, 1R21NS101469

Original Acknowledgements:

We thank Dr. Priyattam Shiromani, Dr. Blanco-Centurion Carlos, and Dr. SiWei Luo for advice, guidance, and technical support on calcium imaging and data processing. We thank Mr. Aurelio Vidal-Ortiz for technical support on animal surgery. Supported by NIH grants 1R01NS096151, 1R21NS101469.

The article has been corrected accordingly.

