# Supplementary material for: Activity dynamics of amygdala GABAergic neurons during cataplexy of narcolepsy
Source: eLife. 2019 Aug 14;8:e48311. doi: 10.7554/eLife.48311 (PMC6703899; doi:10.7554/eLife.48311)
Supplement: Figure 1—source data 1. — Percentages inside the parenthesis in column (A) were calculated as of total GCaMP6 expressing neurons in the amygdala structure. More than 80% of GCaMP6 expressing neurons were located in CeA. About 95% of them also contained VGAT. CeA: central nucleus of the amygdala, BLA: basolateral amygdala, BMA: basomedial amygdala. [file elife-48311-fig1-data1.docx]

**Figure 1-source data**

Numbers of GCaMP6 and VGAT expressing neurons in the amygdala of the narcoleptic mice (n=5)

|  | (A)  GCaMP6+ | (B)  GCaMP6+/VGAT+ | B/A (%) |
| --- | --- | --- | --- |
| CeA | 835 (81.70%) | 793 | 94.97 |
| BLA | 102 (9.98%) | 94 | 92.16 |
| BMA and adjacent areas | 85 (8.32%) | 81 | 95.29 |
